# Effects of Cape Cobra (*Naja nivea*) Venom and Its Isolated Protein on the Modulation of Platelet Activation

**DOI:** 10.3390/toxins18050211

**Published:** 2026-04-30

**Authors:** Mahtab Khatibi, José R. Almeida, Ashifa Al Juwaiser, Soheil Gilabadi, Ketan Patel, Sakthivel Vaiyapuri

**Affiliations:** 1 School of Pharmacy, University of Reading, Reading RG6 6UB, UK; m.khatibi@pgr.reading.ac.uk (M.K.); rafael.dealmeida@ikiam.edu.ec (J.R.A.); a.aljuwaiser@pgr.reading.ac.uk (A.A.J.); s.gilabadi@pgr.reading.ac.uk (S.G.); 2 Biomolecules Discovery Group, Universidad Regional Amazónica Ikiam, Tena 150150, Ecuador; 3 School of Biological Sciences, University of Reading, Reading RG6 6UB, UK

**Keywords:** elapid, cape cobra, *Naja nivea*, haemostasis, blood coagulation, three-finger toxins and cytotoxin

## Abstract

The Cape cobra (*Naja nivea*), one of Africa’s most lethal snakes, can cause rapid, life-threatening paralysis. However, the impact of this venom on platelet function and blood coagulation remains poorly understood. To address this gap, we investigated the enzymatic profiles and the impacts of *N. nivea* venom on multiple aspects of haemostasis using human whole blood. Our results illustrate that Cape cobra venom significantly increases clotting time in rotational thromboelastometry without affecting other coagulation parameters. This venom significantly inhibits platelet aggregation and activation yet does not exert cytotoxic effects on platelets. The venom was subsequently fractionated using reverse-phase high-performance liquid chromatography, and the most potent purified fraction was identified as a cytotoxin (three-finger toxin) through mass spectrometry. This purified fraction showed an inhibitory effect on platelet activity. These findings highlight that *N. nivea* venom can induce haemotoxicity in addition to neurotoxicity. Moreover, three-finger toxins may be promising candidates for bioprospecting to develop novel antithrombotic agents.

## 1. Introduction

Snake venoms are widely recognised for their significant effects on blood haemostasis. In particular, viper venoms contain haemotoxic components that influence haemostasis by either promoting or inhibiting clot formation, thus disrupting the normal clotting process [1,2]. Blood clot formation is a vital physiological process where platelets and plasma proteins, specifically fibrinogen, form a fibrin mesh to stop bleeding and maintain the integrity of the haemostatic system during injury [3,4]. Any disturbance in this balance can result in thrombosis or haemorrhage, leading to various morbidities and mortalities [3,4]. For example, several viper venoms contain procoagulant factors, such as prothrombin activators and thrombin-like proteases, that stimulate the coagulation pathways, increasing fibrin production and depleting clotting factors, a condition known as venom-induced consumption coagulopathy (VICC) [2,5]. Conversely, some viper venoms contain anticoagulant components, such as fibrinolytic enzymes and inhibitors of platelet function and specific clotting factors, which disrupt normal clot formation, resulting in uncontrolled bleeding [1,2,5]. Although snakebite envenomation can lead to serious clinical complications, venom components have also garnered significant interest as molecular tools for clinical diagnosis and treatment of human coagulation disorders [6,7]. Nonetheless, ongoing research into these venom components remains vital for improving clinical management and developing innovative therapeutic strategies for snakebites and other human diseases. To date, research on venom-induced coagulopathy has mainly concentrated on viperid species due to their well-documented haemotoxic effects [8]. In such envenomations, coagulopathy most commonly manifests as VICC, characterised by rapid activation and depletion of clotting factors, resulting in incoagulable blood and a high risk of bleeding. This pathology arises from venoms rich in enzymatic toxins, including snake venom metalloproteases (SVMPs), snake venom serine proteases (SVSPs), and phospholipases A_2_ (PLA_2_s) [9,10].

In contrast, the venoms of elapid species (e.g., cobras, kraits, and mambas) are primarily characterised by non-enzymatic low-molecular-mass components, such as three-finger toxins (3FTXs) [9,10] and PLA_2_, which is why they have been mainly studied for their neurotoxic properties, leading to limited understanding of their potential haemotoxic effects on the coagulation system [8]. Elapid venoms have long been regarded as solely neurotoxic, inducing paralysis with little effect on blood coagulation. However, recent findings challenge this view, suggesting that some elapid snakes contain venom components that can affect haemostatic processes. For instance, an isolated PLA_2_ from the venom of *Naja naja* has been reported to disaggregate platelets and inhibit collagen- and thrombin-induced platelet aggregation in platelet-rich plasma [11]. Several African spitting cobras have been shown to possess toxins with significant anticoagulant effects that inhibit essential clotting factors [8,12]. Given this context, this study aims to explore the effects of the venom of the Cape cobra (*Naja nivea*), an elapid snake, on haemostasis. *N. nivea* is a medically significant cobra species, mainly distributed across South Africa, Botswana, and Namibia [13,14]. This snake is classified as a Category 1 medically important snake by the World Health Organisation (WHO), due to its frequent involvement in human envenomation incidents and the high fatality rates reported in these regions [13,14,15]. Envenomation by the Cape cobra is predominantly neurotoxic, often resulting in rapid development of flaccid paralysis that necessitates immediate respiratory support and antivenom administration, and may be fatal if left untreated [13]. This is because almost 75% of its venom proteome possesses 3FTXs, which can target various molecular receptors, including nicotinic and muscarinic acetylcholine receptors and L-type calcium channels [16,17]. In the Western Cape, clinicians have observed that Cape cobra bites, while less common than other snakebites, result in disproportionately high morbidity and mortality [18].

The rationale of this study is to determine whether *N. nivea* venom contains potential haemotoxic components. The aim is to investigate the in vitro effects of this venom on platelet activation and coagulation cascades. Cape cobra venom exhibits substantial enzymatic and anticoagulant activity, and fractionation has identified a cytotoxin with potent antiplatelet activity. In addition to understanding the pathophysiology of venom, research on such poorly understood venoms offers significant opportunities for bioprospecting, enabling the discovery of therapeutically valuable molecules for human thrombotic conditions and bleeding disorders.

## 2. Results

### 2.1. Cape Cobra Venom Shows Metalloprotease and Phospholipase A_2_ Activities

**Figure 1:**
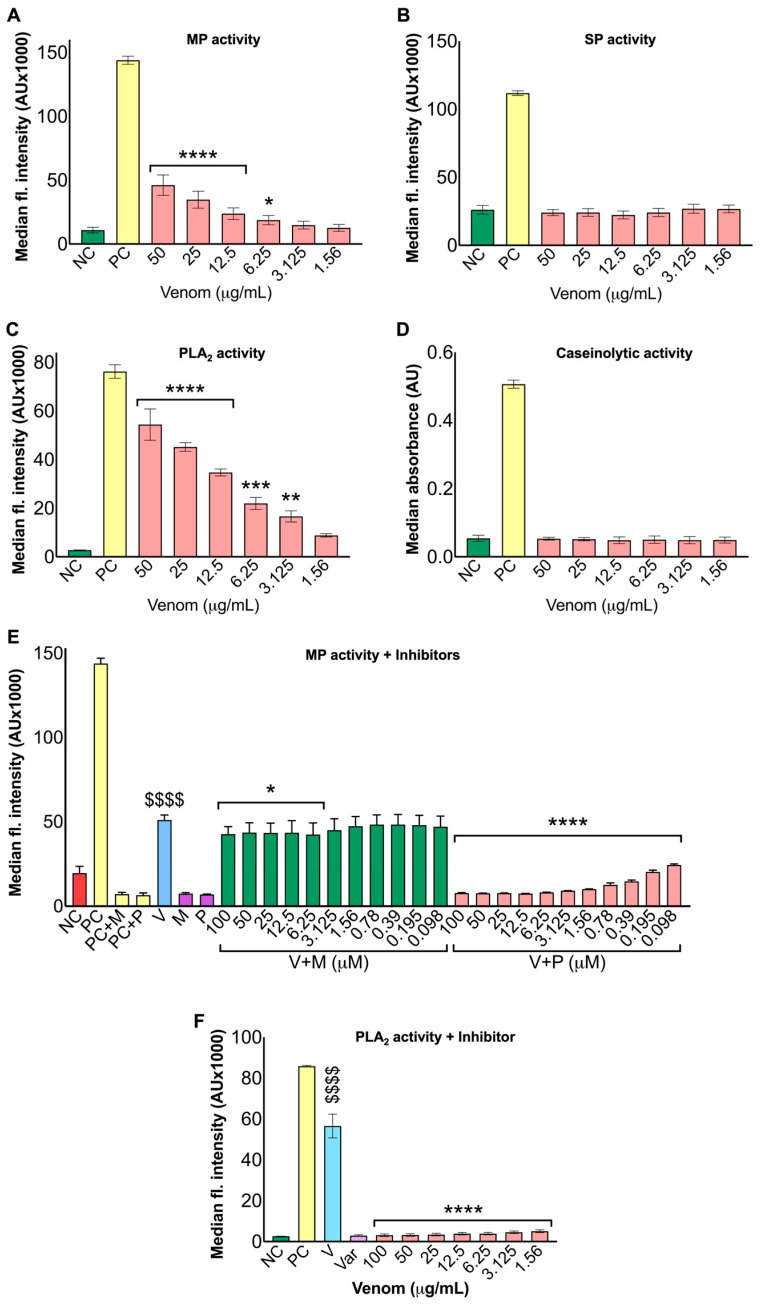
Enzymatic characterisation of *Naja nivea* venom. The metalloprotease activity (MP; (**A**)), serine protease activity (SP; (**B**)), phospholipases A_2_ activity (PLA_2_; (**C**)), and caseinolytic activity (**D**) of various concentrations (50–1.56 μg/mL) of *N. nivea* venom were measured using spectrofluorimetry with suitable fluorogenic or colourimetric chromogenic substrates. (**E**) MP activity was assessed in venom (50 μg/mL), in the presence and absence of various concentrations (100–0.098 μM) of marimastat (M) and prinomastat (P). (**F**) PLA_2_ activity of venom following incubation with varespladib (Var) at concentrations ranging from 100–1.56 μM. For all the assays, *C. atrox* venom (50 μg/mL) and phosphate-buffered saline (PBS) without substrate served as the positive (PC) and the negative (NC) controls, respectively. In (**A**–**D**), all venom-treated samples were compared with the NC to assess statistical significance. In (**E**,**F**), the venom-alone samples (V) were compared to the NC (shown as $), while inhibitor-treated samples were compared to the V (shown as *) to calculate the significance. Data presented mean ± S.D. (*n* = 6). Statistical analysis was performed using one-way ANOVA followed by Fisher’s LSD (* *p* ≤ 0.05, ** *p* ≤ 0.01, *** *p* ≤ 0.001 and **** *p* ≤ 0.0001).

To characterise the enzymatic profile of *N. nivea* venom, metalloprotease (MP), serine protease (SP), phospholipase A_2_ (PLA_2_) and caseinolytic assays were performed using DQ gelatin, BAAMC, a fluorogenic PLA_2_ substrate (Red/Green BODIPY^®^ PC-A2) and azocasein, respectively. The MP assay demonstrated concentration-dependent activity at the highest concentration (50 μg/mL), reaching approximately one-third of the positive control (PC; *Crotalus atrox* venom). MP activity remained significant following a moderate decline at 25 μg/mL but decreased sharply at lower concentrations (3.125–1.56 µg/mL), where values approached those of the negative control (NC), indicating limited MP activity at lower concentrations (Figure 1A). In contrast, the SP (Figure 1B) assay showed no significant activity across all tested venom concentrations, with results similar to those of the NC. The PLA_2_ assay showed significant enzymatic activity, with the highest concentration (50 μg/mL) peaking at approximately 75% of the PC, followed by a gradual reduction in response at lower concentrations (25–3.125 μg/mL) (Figure 1C). The enzymatic activity remained within 40–70% of the (PC) across most concentrations; however, the lowest concentration (1.56 μg/mL) did not elicit a statistically significant response. The caseinolytic (Figure 1D) assay also showed no significant activity across all tested concentrations, indicating either no or minimal proteolytic activity in this venom. These data indicate that *N. nivea* venom exhibits MP and PLA_2_ activities but lacks significant SP and caseinolytic activities at the concentrations evaluated.

To examine the inhibitory effects of marimastat and prinomastat (small-molecule MP inhibitors) on MP activity, and varespladib (a small-molecule PLA_2_ inhibitor) on PLA_2_ activity, a series of assays with various concentrations of inhibitors was performed. For marimastat and prinomastat (100–0.098 μg/mL), the first five marimastat-treated concentrations showed significant inhibition relative to the venom-only (V) sample, whereas the remaining concentrations did not significantly inhibit venom MP activity. All concentrations of prinomastat-treated samples caused a strong, dose-dependent inhibition, with MP activity suppressed to near-baseline at higher concentrations (100–1.56 μg/mL), followed by a gradual recovery toward the final concentration (Figure 1E). For PLA_2_ activity, treatment with varespladib (100–1.56 μg/mL) completely inhibited enzymatic activity, restoring it to baseline and demonstrating statistical significance across the entire concentration range used (Figure 1F). Collectively, these results show the limited efficacy of marimastat, but the potent inhibitory effect of prinomastat against MP activity, and the complete inhibition of PLA_2_ activity by varespladib.

### 2.2. N. nivea Venom Prolongs Clotting Time

**Figure 2:**
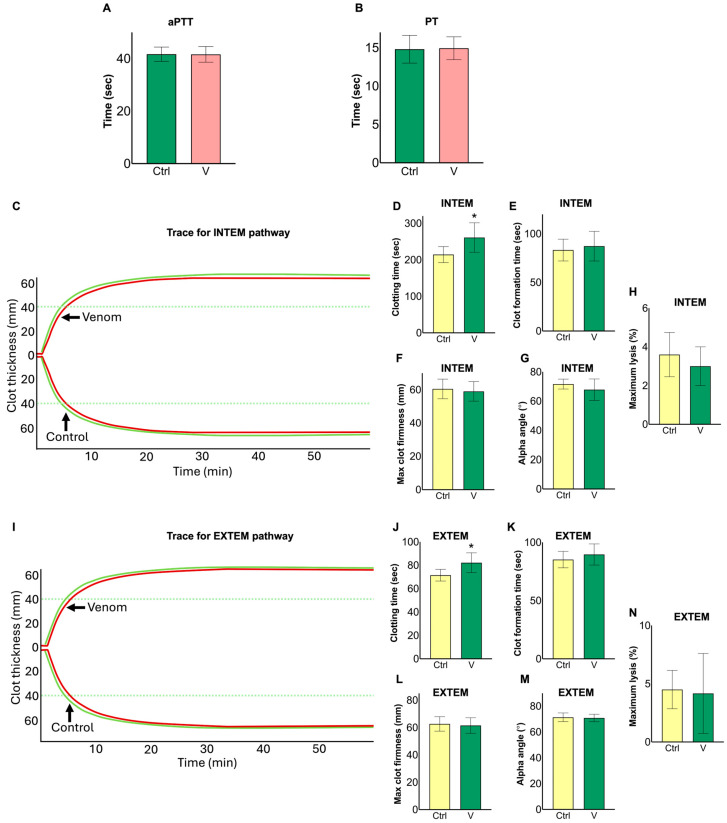
Effect of *N. nivea* venom on coagulation parameters. Activated partial thromboplastin time (aPTT; (**A**)) and prothrombin time (PT; (**B**)) were measured using the Ceveron T100 automated coagulation analyser using citrated human plasma in the presence and absence of venom. Rotational thromboelastometry (ROTEM; (**C**–**N**)) was used in whole blood to assess both the Intrinsic (INTEM) and extrinsic (EXTEM) pathways in the presence and absence of venom. Representative traces for INTEM (**C**) and EXTEM (**I**) are shown. Coagulation parameters, including clotting time (CT; (**D**,**J**)), clot formation time (CFT; (**E**,**K**)), maximum clot firmness (MCF; (**F**,**L**)), alpha angle (**G**,**M**) and maximum lysis (ML; (**H**,**N**)), were measured for both assays. The venom (V) concentration was 50 μg/mL in all experiments, and PBS served as the control (Ctrl). Data represent mean ± S.D. (*n* = 4). Statistical analysis was performed using a *t*-test in GraphPad Prism (* *p* ≤ 0.05).

To investigate the effect of *N. nivea* venom on the intrinsic and extrinsic pathways of the coagulation system, tests for activated partial thromboplastin time (aPTT) and prothrombin time (PT) were performed, respectively, in human plasma. In both assays, the clotting profiles of the venom-treated samples (50 μg/mL) were similar to those of the negative control (Ctrl), indicating that *N. nivea* venom did not affect these measurements under the conditions tested (Figure 2A,B). To assess the potential impact of *N. nivea* venom on whole blood clotting, rotational thromboelastometry (ROTEM) was conducted using the INTEM and EXTEM assays, which target the intrinsic and extrinsic coagulation pathways, respectively. In both pathways, a statistically significant increase (around 25%) in clotting time (CT) compared to the control (Ctrl) was observed following exposure to *N. nivea* venom (50 μg/mL), indicating a delay in the initial phase of clot formation (Figure 2C,D,I,J). However, no changes were detected in other thromboelastometry parameters, including clot formation time (CFT), maximum clot firmness (MCF), alpha angle (α-angle), and maximum lysis (ML), all of which remained comparable to the Ctrl (Figure 2E–H,K–N). These findings suggest that *N. nivea* venom may influence the initial phase of clotting, potentially extending the onset of clot formation in both pathways, but does not affect other parameters regulating clot development or lysis within the intrinsic, extrinsic, or common pathways.

### 2.3. N. nivea Venom Suppresses Platelet Activation

**Figure 3:**
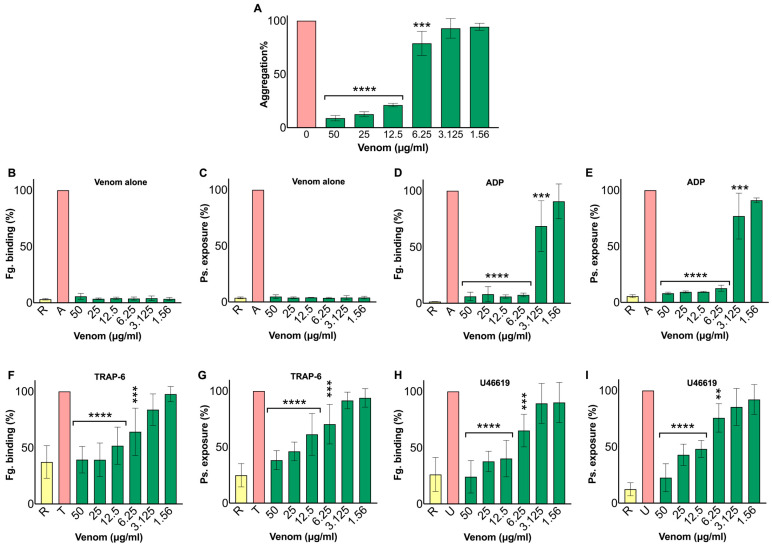
Effect of *N. nivea* venom on platelet aggregation and activation. (**A**) Platelet aggregation was measured using an optical aggregometer with platelet-rich plasma (PRP) and adenosine diphosphate (ADP; 5 μM) as an agonist to test various venom concentrations (12.5–1.56 μg/mL). Aggregation activity for all venom concentrations in the presence of ADP is shown. (**B**–**I**) Flow cytometry was used to assess fibrinogen binding and P-selectin exposure following venom addition. Different venom concentrations (50–1.56 μg/mL) were tested, either alone (**B**,**C**) or combined with platelet agonists such as ADP (5 μM) (**D**,**E**), TRAP-6 (10 μM) (**F**,**G**), and U46619 (4 μM) (**H**,**I**). Resting (R; PRP alone) platelets served as the baseline, while samples treated with agonists only were set to 100% to normalise the results. Data are expressed as mean ± S.D. (*n* = 4). Statistical analysis involved one-way ANOVA followed by Fisher’s LSD (** *p* ≤ 0.01, *** *p* ≤ 0.001 and **** *p* ≤ 0.0001).

To evaluate the impact of *N. nivea* venom on platelet aggregation, an optical aggregometry assay was performed using adenosine diphosphate (ADP, 5 μM) as an agonist. During the initial incubation phase (pre-agonist), no platelet aggregation was observed in any of the samples with venom, indicating that the venom did not induce aggregation on its own. After the addition of the agonist, venom-treated samples showed concentration-dependent inhibition of aggregation compared to the ADP control (0). Samples containing 12.5 and 6.25 μg/mL venom concentrations exhibited significant reductions in response, with platelet aggregation decreasing to less than 25% and 75% of the ADP control (0), respectively. Conversely, at 3.125 and 1.56 μg/mL venom concentrations, the inhibitory effect was less evident, with responses similar to the ADP control (0), suggesting no or minimal inhibition at these lower venom concentrations (Figure 3A). Overall, the results indicate that *N. nivea* venom markedly inhibits platelet aggregation at mid-range concentrations compared to the ADP control.

To further investigate the effect of *N. nivea* venom on platelet activation, flow cytometry was utilised to assess fibrinogen binding and P-selectin exposure as markers of platelet activation under resting and agonist-stimulated conditions. Exposure to the venom alone did not influence either activation marker, indicating no activity on its own on platelets (Figure 3B,C). In contrast, with agonists such as ADP (5 μM), thrombin receptor activating peptide-6 (TRAP-6, 10 μM), and U46619 (4 μM), higher venom concentrations (50–12.5 μg/mL) caused a significant dose-dependent reduction in platelet activation, with levels of both markers decreasing by 25–80% compared to agonist-only controls. Responses approached baseline (100%) at lower venom concentrations (6.25–1.56 μg/mL) (Figure 3D–I). These findings emphasise that *N. nivea* venom can inhibit platelet activation by targeting either ADP receptors, thrombin pathways, or thromboxane signalling, or their common pathways, thereby suppressing integrin αIIbβ3 activity and α-granule secretion.

### 2.4. N. nivea Venom Degrades the Aα Chain of Fibrinogen

**Figure 4:**
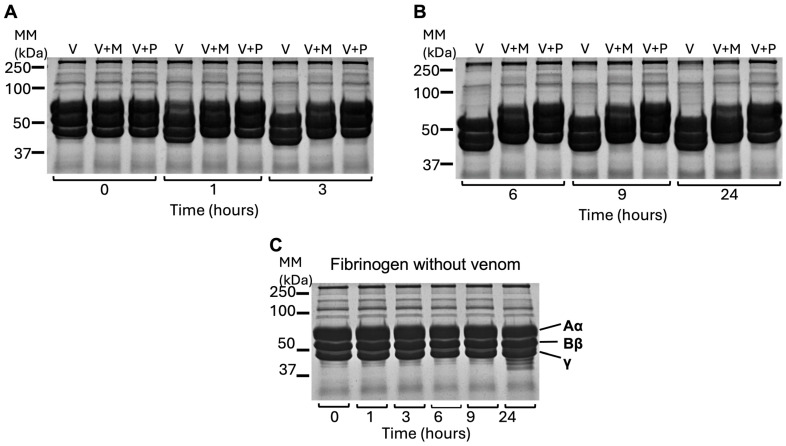
Fibrinogenolytic activity of *N. nivea* venom. SDS-PAGE gel electrophoretic profiles (12% gel) show the fibrinogenolytic activity of *N. nivea* venom incubated with human fibrinogen (final concentration of 1 mg/mL) at 37 °C for various time points ((**A**); 0, 1, 3 h) and ((**B**); 6, 9, 24 h). The activity was examined in venom alone (V) and in venom with marimastat (V+M) and prinomastat (V+P). Following incubation with human fibrinogen, samples were collected at each time point, analysed by SDS-PAGE, and protein bands were visualised using Coomassie staining. (**C**) Fibrinogen incubated without venom was used as a reference to demonstrate the stability of fibrinogen in the absence of venom and to facilitate comparison with venom-treated samples. The positions of the fibrinogen Aα, Bβ, and γ chains are indicated on the reference gel. MM represents the molecular mass marker.

To assess the fibrinogenolytic activity of *N. nivea* venom, human fibrinogen was incubated with the venom at specified time points, with and without marimastat and prinomastat, and then analysed by SDS-PAGE. At the initial time point (0 h), all conditions, including venom alone (V) and inhibitor-treated samples (V+M) and (V+P), displayed similar protein band profiles, indicating no early chain degradation. In the early mid-phase (1–3 h), the venom-treated samples showed progressive degradation of the Aα chain. Marimastat-treated samples (V+M) showed moderate digestion, while prinomastat-treated samples (V+P) fully preserved the bands’ integrity (Figure 4A). During the extended phase (6–24 h), venom-only samples exhibited complete degradation of the Aα chain, while marimastat showed mild inhibition, and prinomastat maintained full Aα chain integrity (Figure 4B). Throughout the assay, the Bβ chain remained intact, indicating selectivity of this venom towards the Aα chain. Fibrinogen-only controls showed no digestion at any time point, confirming the stability of fibrinogen in the absence of venom (Figure 4C). Overall, these results demonstrate that *N. nivea* venom exerts a selective, time-dependent fibrinogenolytic effect, with marimastat partially suppressing and prinomastat fully blocking this activity.

### 2.5. N. nivea Venom Does Not Exert Haemolytic Activity

To evaluate the haemolytic activity of *N. nivea* venom, washed human erythrocytes were incubated with the venom at 37 °C for 0, 1, 6, and 24 h. Under all tested conditions, *N. nivea* venom showed no detectable haemolytic activity, with responses remaining similar to the negative control, regardless of the presence of prinomastat or varespladib (Figure S1A–E). Since no significant erythrocyte lysis was observed, the addition of inhibitors did not alter the response. These findings indicate that *N. nivea* venom exhibits no or negligible haemolytic activity.

### 2.6. A Three-Finger Toxin from N. nivea Venom Inhibits Platelet Activation and Fibrinogen Binding

**Figure 5:**
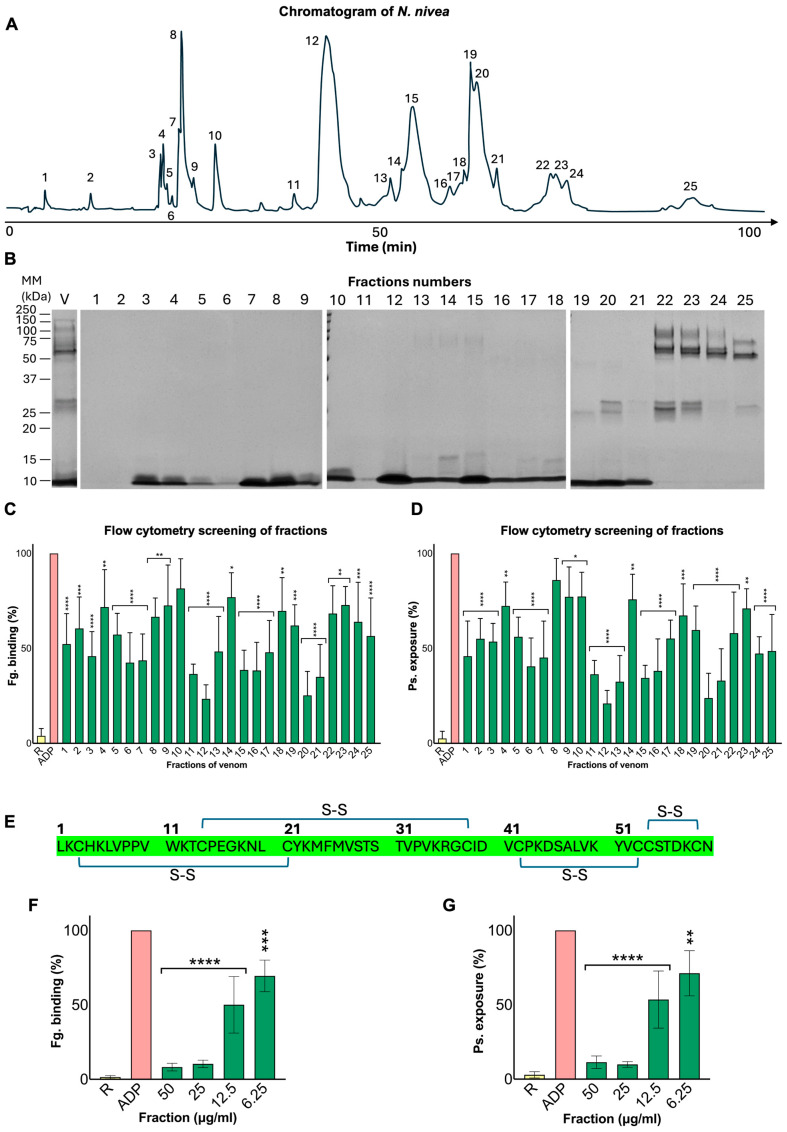
Purification and characterisation of a cytotoxin from *N. nivea* venom. (**A**) Reversed-phase high-performance liquid chromatography (RP-HPLC) chromatogram of crude venom. 2 mg of *N. nivea* venom was loaded onto a C18 column, and 25 fractions were collected over 100 min. (**B**) SDS-PAGE analysis (12% gel) of all collected fractions, showing distinct protein bands. MM represents the molecular mass marker. The level of (**C**) fibrinogen binding and (**D**) P-selectin exposure on platelets in the presence of each collected fraction (5 µL of each fraction) was assessed using flow cytometry. All activities were normalised to the levels of fibrinogen binding and P-selectin exposure obtained with ADP alone (shown as ADP), which was considered as 100%, and statistical analysis of each fraction was measured against this control. (**E**) Protein sequence of fraction 12, identified as cytotoxin (3FTX). Green highlights show the matching sequences to an existing *N. nivea* cytotoxin’s sequence in the Uniprot database. Fibrinogen binding (**F**) and P-selectin exposure (**G**) of various concentrations of the purified fraction were tested using ADP (5 μM). Resting platelets (R; PRP alone) were considered the baseline, whereas ADP was set as 100% and used as the reference to normalise the results. Data are expressed as mean ± S.D. (*n* = 4). Statistical analysis involved one-way ANOVA followed by Fisher’s LSD (* *p* ≤ 0.05, ** *p* ≤ 0.01, *** *p* ≤ 0.001 and **** *p* ≤ 0.0001).

To isolate proteins with platelet-inhibitory effects from *N. nivea* venom, reverse-phase high-performance liquid chromatography (RP-HPLC) was conducted using a C18 column, collecting 25 fractions over a 100-min gradient. The prominent peaks in the centre of the chromatogram suggest the dominance of hydrophobic components, such as cytotoxins, over polar components like MPs (Figure 5A). Subsequent SDS-PAGE analysis of the collected fractions revealed concentrated protein bands around 10 kDa, particularly in fractions 12–21. Additional bands with higher molecular masses were also observed in later fractions (fractions 22–25), indicating the presence of other venom components with larger molecular masses (Figure 5B). Flow cytometric assays showed a significant reduction in P-selectin exposure and fibrinogen binding in nearly all fractions, with fraction 12 exhibiting the most potent inhibition (Figure 5C,D). This specific fraction also exhibited a sharp peak in the chromatographic profile and a distinct single band on SDS-PAGE. Consequently, fraction 12 was chosen for mass spectrometry analysis, in which peptide fingerprinting confirmed it as a venom-derived three-finger toxin (3FTX), sharing 100% identity with *N. nivea* venom’s cytotoxin 1 (UniProt accession no. P01456) (Figure 5E).

To further explore the effect of the purified protein on platelet reactivity, several concentrations of the protein (50–6.25 μg/mL) were prepared and tested using flow cytometry with ADP as an agonist. The results confirmed the ability of this fraction to significantly inhibit both P-selectin exposure and fibrinogen binding in platelets in a dose-dependent manner (Figure 5F,G). In this experiment, the activity of both parameters decreased to approximately 10% at concentrations of 50 and 25 μg/mL of the isolated protein, with partial recovery at 12.5 μg/mL (almost 50%) and 6.25 μg/mL (almost 70%) compared to the positive control. Overall, these findings suggest that fraction 12 is a pure cytotoxin or 3FTX with a significant inhibitory effect on platelet reactivity.

### 2.7. N. nivea Venom and Its Purified 3FTX Are Cytotoxic for AB1190 Cells but Not for Platelets

**Figure 6:**
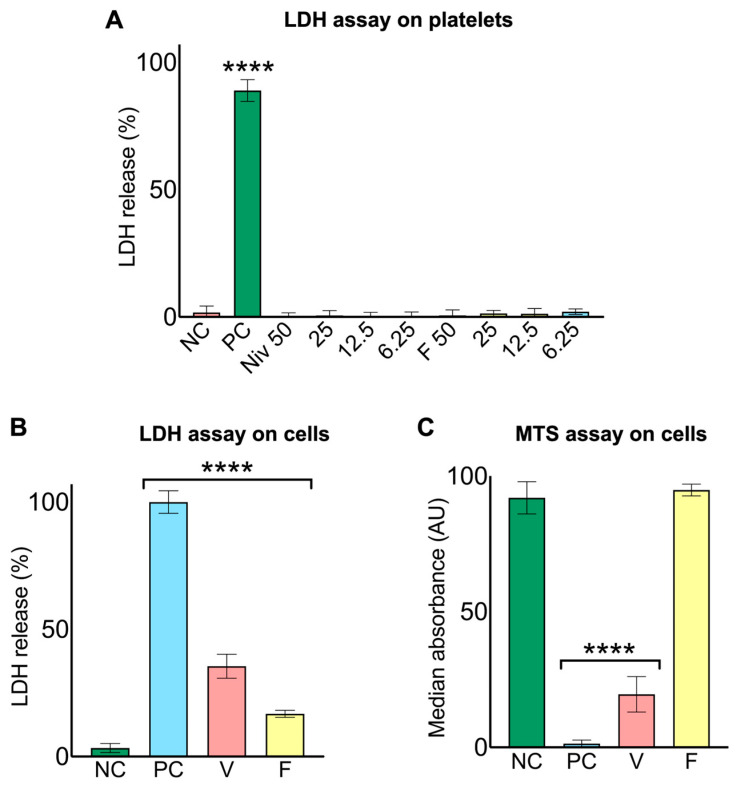
Cytotoxic effects of *N. nivea* venom. The cytotoxicity effect of *N. nivea* venom was assessed using the LDH and MTS assay. (**A**) LDH release from washed human platelets was measured following exposure to both *N. nivea* venom and its purified protein at four different concentrations (50–6.25 μg/mL) using CyQUANT™ LDH Cytotoxicity kit (Thermo Fisher Scientific, Waltham, MA, USA). (**B**) LDH activity of *N. nivea* venom (50 μg/mL) and its purified protein (50 μg/mL) on undifferentiated human myoblast cells (AB1190) was measured using the same LDH assay kit. (**C**) Impact of *N. nivea* venom (50 μg/mL) and its purified protein (50 μg/mL) on the viability of AB1190 myoblasts was assessed using the MTS assay. For all assays, the absorbance of samples was measured at 490 and 680 nm using a plate reader. A detergent [1% (*v*/*v*) of Triton X-100] was used as the positive control (PC). The data are represented as percentages relative to the controls. For statistical analysis, all samples were compared against the negative control (NC; PBS). Data is represented as mean ± S.D. (*n* = 3). Statistical analysis involved one-way ANOVA followed by Fisher’s LSD (**** *p* ≤ 0.0001).

To evaluate the cytotoxicity of *N. nivea* venom and its purified protein, the lactate dehydrogenase (LDH) assay was conducted on human washed platelets. Results showed that neither the *N. nivea* venom nor its purified cytotoxin caused significant LDH release, as all tested concentrations (50–6.25 µg/mL) were comparable to the negative control, indicating no cytotoxicity towards platelets (Figure 6A). Conversely, when the same experiment was performed on cultured AB1190 human myoblast cells, both the venom and its purified protein elicited minimal toxicity, likely reflecting the greater susceptibility of nucleated muscle cells to membrane-disruptive cytotoxins compared with anucleated platelets. Exposure to the whole venom sample resulted in nearly 40% LDH release, while the purified protein caused a modest response of around 20%. Both increases were significantly higher than the negative control but lower than the positive control, which only had the detergent to exert maximum cytotoxicity (Figure 6B). To confirm these findings and assess their impact on cell viability, an MTS assay was also performed on AB1190 cells. While the negative control maintained full metabolic activity (100%), the positive control nearly abolished cell viability. Cells treated with *N. nivea* venom (50 µg/mL) showed reduced viability to almost 25%, whereas those treated with the purified protein (50 µg/mL) retained viability at nearly baseline levels (Figure 6C). These results demonstrate that *N. nivea* venom’s cytotoxicity is specific to certain cells, such as myoblasts, with platelets largely unaffected, and components other than the purified protein may be responsible for the cytotoxic effects observed in these experiments.

## 3. Discussion

Envenomations by cobra snakes are a major health concern, as several *Naja* species can result in fatal outcomes [13,14,19]. Unlike viper venoms, which are largely recognised for their severe haemotoxicity through metalloproteases, serine proteases, and other prothrombotic toxins that directly activate clotting factors such as prothrombin, Factor X, and V [5,20], cobra venoms are predominantly neurotoxic, leading to rapid flaccid paralysis and respiratory failure [13,21]. As a result, most research on elapid snake venoms to date has focused on neurotoxicity, while their haemotoxicity has received less attention [8]. However, some studies indicate that certain cobra venoms can significantly interfere with the coagulation system. For instance, African spitting cobras contain potent anticoagulant components that inhibit key clotting factors, including Factor Xa and thrombin, thereby impairing clot formation [8]. These cobras have been linked to at least one death caused by severe bleeding following envenomation [8]. In contrast, non-spitting cobras such as the Cape cobra (*N. nivea*) are primarily neurotoxic and are among the most clinically significant African snakes, capable of causing life-threatening paralysis within a few hours of envenomation [13]. Epidemiological records from South Africa’s Western Cape report that only 14 Cape cobra bite cases were recorded over five years (out of 122 snake envenomations reported), but *N. nivea* was responsible for causing the most morbidity and mortality in that area [18]. Data on the haemotoxic effects of this venom are limited; existing studies mainly involve proteomic analyses reporting low levels of PLA_2_ and snake venom metalloproteases (SVMPs), consistent with our findings, but also highlight three-finger toxins (3FTXs), such as cytotoxins (75.6%) and alpha-neurotoxins (around 7.4%), as the dominant venom components which can explain the clinical pattern of profound systemic paralysis with minimal local injury [13,14,22]. Furthermore, specific antivenom treatments for this venom are limited [13]. South Africa’s standard polyvalent antivenom (SAIMR Polyvalent) includes Cape cobra venom in the immunising mix; however, evidence suggests that even this specific antivenom may be limited in its ability to neutralise Cape cobra venom once severe envenoming has occurred [13]. In cases of Cape cobra envenomation, apart from a few reports of mild alterations in coagulation parameters and increased fibrin degradation, significant coagulation impairments have not been observed [23]. Therefore, we assessed the potential haemotoxic effects of Cape cobra venom, as this remains poorly understood.

Based on our findings, *N. nivea* venom significantly inhibits human platelet aggregation and activation without causing cytotoxic effects on platelets. It markedly diminishes platelet aggregation induced by adenosine diphosphate (ADP) at high concentrations and inhibits platelet activation in a dose-dependent manner. Both markers of platelet activation, fibrinogen binding and P-selectin exposure, were reduced following exposure to different concentrations of venom upon activation with agonists such as ADP, TRAP-6, and U46619. These findings suggest that the antiplatelet activity of *N. nivea* venom results from a specific pharmacological mechanism rather than cytotoxic effects on platelets. This aligns with studies on other cobra venoms; for example, a purified 3FTX from *N. kaouthia* venom inhibited platelet aggregation following the addition of ADP, thrombin, and arachidonic acid agonists [24]. Furthermore, PLA_2_, a lipid-hydrolysing enzyme that cleaves membrane phospholipids to generate bioactive mediators, is a potent inhibitor of human platelet aggregation in *N. nigricollis* venom [25]. The same effect is observed in other elapid venoms, such as *Notechis scutatus*, which also inhibits human platelet aggregation [26]. Conversely, viper venoms are predominantly procoagulant due to high levels of prothrombin activators and other coagulation enzymes, including metalloproteases (MPs) and serine proteases (SPs) [27]. *Daboia russelii* venom exemplifies this, with strong procoagulant components that cause extensive clotting (fibrin formation) via a metalloprotease-derived Factor X activator (RVV-X), which then converts prothrombin to thrombin. Despite this, some viper venoms contain anticoagulant constituents; for instance, *Echis carinatus* venom contains echistatin, a small disintegrin that binds to platelet integrin GPIIb/IIIa, thereby inhibiting platelet aggregation [28]. Overall, the platelet-inhibitory effect of *N. nivea* aligns with previously reported findings of other cobra and elapid venoms.

To distinguish platelet-specific effects from direct modulation of the coagulation cascade, plasma and whole blood clotting assays were performed. Results showed that *N. nivea* venom did not significantly alter standard plasma clotting times (PT or aPTT), indicating no notable effect on the extrinsic or intrinsic pathways under the tested conditions. Conversely, *N. nivea* venom slightly prolonged clotting time in both the intrinsic and extrinsic pathways during ROTEM assays with whole blood, while other thromboelastometry parameters remained unchanged. This pattern suggests that *N. nivea* venom may exert a mild anticoagulant effect during the initiation phase of coagulation, without affecting clot formation or strength. African spitting cobra venoms, such as *N. nigricolis*, *N. nigricincta*, *N. pallida*, and *N. mossambica*, inhibit Factor Xa via PLA_2_ toxins, impairing prothrombinase complex formation and delaying clot initiation [12]. Conversely, certain elapid venoms act as potent procoagulants. For instance, *Oxyuranus scutellatus* venom contains a prothrombin activator that converts prothrombin into thrombin, leading to consumptive coagulopathy [29]. *Pseudonaja textilis* venom also causes similar effects through pseutarin C, a prothrombin activator [30]. In contrast, viper venoms employ a markedly different mechanism to induce coagulotoxic effects. For example, specific viper venoms, such as *Bothrops atrox*, *Echis ocellatus*, and *D. russelii*, have strong procoagulant effects, directly activating clotting factors via SVMP and SVSP enzymes [31,32]. *Bothrops* species possess thrombin-like serine proteases that facilitate fibrinogen clotting, as well as specific enzymes that activate factor X [4,33]. Similarly, *E. ocellatus* venom uses its SVMP to activate both prothrombin and factor X [31]. *D. russelii* venom contains enzymes that activate factor V and X, ultimately leading to consumptive coagulopathy [32]. For example, 3FTXs from elapid venoms have been characterised as interacting with a variety of cardiovascular targets, including platelet receptors and ion channels, suggesting various mechanisms by which they can influence haemostasis and vascular function, distinct from direct enzymatic activation of coagulation factors used by viper venoms [34].

Fractionation of crude *N. nivea* venom via RP-HPLC yielded 25 distinct fractions. Among these, one abundant fraction (F12), which showed the greatest inhibitory effect on platelet activation in flow cytometry assays, was identified as a cytotoxin, a 3FTX, through mass spectrometry. 3FTXs are a structurally conserved family of proteins comprising nearly 60 amino acids. These small polypeptides are stabilised by 4–5 disulphide bonds and can be divided into more than 24 subgroups. They are major components of elapid/cobra venoms and are primarily responsible for neurotoxicity or myotoxicity [35,36]. They can also impair haemostasis by targeting various stages of blood clotting, although this effect remains inadequately studied [36]. Our findings provide new evidence that, when tested on isolated platelets at various concentrations, F12 significantly inhibited fibrinogen binding and P-selectin exposure in a dose-dependent manner, consistent with the activity of the crude venom. This fraction did not cause platelet lysis and only slightly compromised the cellular integrity of human myoblasts, which released LDH but remained metabolically active and exhibited normal morphology. Previous studies have reported alterations in platelet aggregation or coagulation pathways induced by non-enzymatic venom components [20,35]. For example, *N. nigricollis* venom contains a cytotoxin that exerts both antiplatelet and anticoagulant effects [35]. KT-6.9, a 3FTX from *N. kaouthia*, has previously demonstrated significant inhibition of ADP-induced aggregation by binding to platelets [24]. *Hemachatus haemachatus* venom contains ringhalectin and exactin, 3FTXs, which can block factor X activation by the extrinsic tenase complex, thereby inhibiting fibrin clot formation [35]. *Dendroaspis angusticeps* produces thrombostatin, a 3FTX that inhibits fibrinogen binding and platelet aggregation by antagonising glycoprotein alpha-IIb/beta-3 [35]. Overall, these published findings align with our results and support the hypothesis that this purified 3FTX can influence thrombosis and inhibit platelet activity through a distinct, non-enzymatic mechanism, likely by binding to platelet receptors and coagulation factors. Further research is necessary to elucidate the broader haemotoxic effects and the detailed mechanism of action of this toxin.

This study has some limitations, including its restriction to in vitro and ex vivo assays, and the detailed molecular interactions and in vivo effects of this toxin remain to be elucidated. Further in vivo investigations would be necessary to determine whether the platelet-inhibitory effects observed in vitro translate into measurable alterations in haemostasis during envenoming. Future research should focus on comprehensive mechanistic investigations to identify the precise targets of the purified toxin. It could also investigate the molecular mechanisms underlying the observed inhibition of platelet activation, including potential modulation of integrin αIIbβ3 activation. Future studies could also include assessment of mitochondrial function in isolated platelets to further characterise the cytotoxic effects of the venom on platelets. Employing fluorescence-based approaches with confocal microscopy and flow cytometry, using antivenom or toxin-specific antibodies, could also help determine the precise venom components that directly bind to platelets. In addition, evaluating platelet aggregation with different agonists (e.g., collagen or epinephrine) could help clarify the specific pathways underlying the observed platelet inhibitory activity. Additionally, further characterisation of the proteolytic components of *N. nivea* venom using more physiologically relevant substrates and techniques, such as gelatin zymography, may help identify the molecular characteristics of the proteases contributing to the observed enzymatic activity. Furthermore, future studies could investigate the venom’s activity against physiological substrates, such as extracellular matrix proteins (e.g., laminin, collagen, and fibronectin), to better understand its potential tissue-related effects. The pharmacological profile of this toxin requires optimisation, including improvements in efficacy, specificity, and safety, to facilitate its development as a novel therapeutic agent.

In conclusion, although cobra envenomation is mainly neurotoxic [25], our findings highlight this venom’s ability to interfere with platelet activity and the coagulation pathway. This effect is notably less strong than that of vipers. It also emphasises the discovery of a 3FTX in Cape cobra venom that inhibits platelet function. 3FTXs are small, rigid, and highly stable structures, making them suitable for protein engineering, therapeutic uses in bioprospecting, and drug development for conditions such as bleeding disorders and thrombotic complications [37]. For example, by reducing the cytotoxic effects of 3FTXs while preserving their anti-platelet activity, these toxins could inspire the development of improved antithrombotic agents, similar to how viper venom disintegrins contributed to the development of tirofiban and eptifibatide [38]. However, many cobra 3FTXs are also known to possess neurotoxic properties [9]; therefore, their potential therapeutic applications would require careful modification and evaluation of their safety profiles. Hence, even though *N. nivea* venom has minimal haemotoxic effects, its components may provide useful insights into mechanisms involved in thrombotic and bleeding disorders. Our findings also suggest that the purified protein may contribute to the venom’s antiplatelet activity without damaging platelet membranes, likely by impairing signalling pathways rather than enzymatically degrading targets. However, this effect appears less potent than that of crude venom, suggesting that other components may act together to produce a stronger haemotoxic response.

## 4. Materials and Methods

Unless otherwise stated, all chemicals and reagents used in this study were of analytical grade and purchased from Sigma-Aldrich (Gillingham, UK).

### 4.1. Venom Preparation

Lyophilised *N. nivea* venom from a pooled stock of multiple specimens was obtained from Latoxan (Valence, France). The lyophilised venom was stored at −80 °C until use. A stock solution was prepared at 2 mg/mL in phosphate-buffered saline (PBS) and stored at −20 °C. On the day of each experiment, the venom stock was freshly diluted in PBS to the required concentrations. During experimental procedures, venom samples were kept on ice to minimise protein degradation. *Crotalus atrox* venom was obtained in lyophilised form from the Kentucky Reptile Zoo (Bowen, KY, USA) and used as a positive control in selected assays. This venom was prepared under the same conditions as *N. nivea* venom, and a concentration of 50 μg/mL was used in relevant assays.

### 4.2. Metalloprotease Assay

The metalloprotease activity of *N. nivea* venom was assessed using DQ-gelatin (1 mg/mL; ThermoFisher Scientific, Abingdon, UK) as a fluorogenic substrate [39]. Several concentrations of venom (50–1.56 μg/mL) were added to a black 96-well plate. Then, 2 μL (2 μg) of DQ-gelatin was added to each well, and PBS was added to bring the final volume to 100 μL in a 96-well microplate. Then, the fluorescence level was recorded at 10-min intervals for 90 min using a plate reader (FLUOstar OPTIMA, Ortenberg, Germany) with excitation and emission wavelengths of 485 nm and 520 nm, respectively. To assess the inhibitory effects of metalloprotease inhibitors, marimastat (100–0.098 μM) and prinomastat (100–0.098 μM) were added to *N. nivea* venom (50 μg/mL) and incubated for 10 min at room temperature in a 96-well plate prior to the addition of DQ-gelatin. Fluorescence was then measured as described above to assess activity. Substrate in PBS and *C. atrox* venom (50 μg/mL) were used as the negative and positive controls, respectively, in the same order.

### 4.3. Serine Protease Assay

Serine protease activity was evaluated using Nα-benzoyl-L-arginine 7-amido-4-methylcoumarin hydrochloride (BAAMC; Sigma Aldrich, Gillingham, UK) as the fluorogenic substrate at a final concentration of 20 μM, reconstituted in Dimethyl sulfoxide (DMSO) [40]. The substrate was added to different venom concentrations (50–1.56 μg/mL) in a black 96-well plate. Then, fluorescence was measured at 10-min intervals for 90 min, using excitation and emission wavelengths of 355 nm and 460 nm, respectively. A Fluostar Optima (BMG Labtech, Ortenberg, Germany) spectrofluorometer was used for this assay. Substrate in PBS and *C. atrox* (50 μg/mL) venom were used as the negative and positive control, respectively.

### 4.4. Phospholipase A_2_ Assay

Phospholipase A_2_ (PLA_2_) activity was evaluated using the Enzcheck^®^ PLA_2_ assay kit (Invitrogen, Paisley, UK), which employed Red/Green BODIPY^®^ PC-A2 as the substrate [39]. This experiment was carried out in black 96-well plates by adding 5 μL of venom, at various concentrations (50–1.56 μg/mL), to 45 μL of reaction buffer, followed by 50 μL of substrate mix (prepared according to the manufacturer’s protocol). Fluorescence readings were taken at 10-min intervals for 90 min using a Fluostar Optima (BMG Labtech, Ortenberg, Germany) spectrofluorometer, set at excitation and emission wavelengths of 485 nm and 520 nm, respectively. Bee venom and substrate in PBS served as the positive (PC) and negative (NC) controls, respectively.

### 4.5. Caseinolytic Assay

Casein was used as a general substrate to assess the venom’s overall proteolytic activity. It is widely used in protease assays due to its stability, low cost, and suitability for detecting broad proteolytic activity. Therefore, this assay was included as a complementary approach to evaluate general protease activity in the venom. The caseinolytic activity was assessed using azocasein (Sigma-Aldrich, Gillingham, UK) as the substrate [41]. The substrate solution was prepared by dissolving 5 mg of substrate in 1 mL of Tris-HCl buffer (50 mM, pH 8.0). The same buffer was used to dissolve the venom and prepare a final concentration of 1 mg/mL. The samples were prepared by mixing 10 μL of venom (50–1.56 μg/mL) with 90 μL of the substrate solution, followed by incubation for 90 min at 37 °C. After incubation, the reactions were halted by adding 200 μL of 5% (*v*/*v*) trichloroacetic acid (TCA). The samples were then centrifuged for 5 min at 8000 rpm at room temperature, and 150 μL of the supernatant was transferred into a 96-well microplate, followed by the addition of 150 μL of NaOH (0.5 M). Absorbance of each well was recorded at 440 nm using a Fluostar Optima (BMG Labtech, Ortenberg, Germany) spectrofluorometer.

### 4.6. Human Blood Collection and Preparation of Plasma, PRP and Washed Platelets

The human blood collection, platelet preparation, and functional assays were performed as previously described [42]. Blood samples were obtained from healthy human donors in accordance with the protocol approved by the University of Reading Research Ethics Committee (UREC 17/17). After acquiring written informed consent, blood was drawn via venepuncture into vacutainers containing 3.25% (*w*/*v*) sodium citrate as an anticoagulant. For platelet-rich plasma (PRP), whole blood was centrifuged at 100× *g* for 20 min at 20 °C. The upper layer was transferred to a 15 mL Falcon tube without disturbing the remaining layers and then maintained at 30 °C before use. Plasma was prepared by centrifuging whole blood at 5000× *g* for 10 min at 20 °C, after which the clear top layer was collected and used in assays.

To isolate platelets, PRP was combined with 3 mL of acid-citrate-dextrose [ACD; 2.5% (*w*/*v*) sodium citrate dihydrate, 2.0% (*w*/*v*) glucose, and 1.5% (*w*/*v*) citric acid] and 10 μL of prostaglandin I_2_ (PGI_2_; 125 μg/mL; Sigma-Aldrich, Gillingham, UK) dissolved in Ethanol. The mixture was gently inverted and centrifuged at 1400× *g* for 10 min at 20 °C. The resulting platelet pellet was resuspended in 1 mL of Tyrode’s-HEPES buffer [134 mmol/L NaCl, 2.9 mmol/L KCl, 0.34 mmol/L Na_2_HPO_4_·12H_2_O, 12 mmol/L NaHCO_3_, 20 mmol/L HEPES, 1 mmol/L MgCl_2_, and 5 mM glucose; pH 7.3], followed by the addition of ACD (150 μL). A volume of 25 mL was achieved by adding pre-warmed Tyrode’s-HEPES buffer along with 150 μL ACD. A second centrifugation was performed after adding 3 mL of ACD and prostacyclin (10 ng/mL) at 1400× *g* for 10 min at 20 °C. The supernatant was discarded, and the final platelet pellet was resuspended in modified Tyrode’s-HEPES buffer to a concentration of 4 × 10^8^ platelets/mL.

### 4.7. Clotting Assay

The Ceveron T100 fully automated coagulation analyser (Technoclone, Vienna, Austria) was used to measure Prothrombin Time (PT) and activated Partial Thromboplastin Time (aPTT) in accordance with the manufacturer’s instructions and as described previously [43]. *N. nivea* venom (50 μg/mL) was added to a fixed volume (40 μL) of human plasma taken from various donors. Standard PT (tissue thromboplastin and 25 mM CaCl_2_) and aPTT (silica/sulfatide phospholipids and 25 mM CaCl_2_) reagents were added following specific protocols, and samples were analysed using Ceveron T100. PT and aPTT measurements were obtained at wavelengths of 405 nm and 630 nm, respectively.

### 4.8. ROTEM Analysis

Rotational Thromboelastometry (ROTEM) analysis was carried out using the ROTEM Delta instrument (Werfen, Warrington, UK) to measure the effect of *N. nivea* venom (50 μg/mL) on various aspects of the whole blood clotting process [43]. A venom concentration of 50 μg/mL was selected based on preliminary experiments and concentrations commonly used in venom functional assays to evaluate potential effects on coagulation parameters. The INTEM and EXTEM tests were carried out to evaluate the impact of the venom on the intrinsic and extrinsic (as well as common) pathways of blood coagulation. In each test, the venom (50 μg/mL) was mixed with citrated human whole blood (300 μL), followed by the addition of specific reagents in predetermined volumes as per the manufacturer’s guidelines. Recalcification of the blood samples was carried out using the star-tem reagent (0.2 M CaCl_2_ in HEPES buffer, pH 7.4). Subsequently, blood coagulation was activated by intrinsic stimulators (partial thromboplastin phospholipid derived from rabbit brain and ellagic acid activators) or extrinsic stimulators (recombinant tissue factor, heparin and phospholipids).

### 4.9. Flow Cytometry and Platelet Aggregation Assays

Platelet aggregation was analysed using optical aggregometry (Chrono-Log, Havertown, PA, USA) [42,43]. The level of aggregation was recorded for 5 min after adding various concentrations of *N. nivea* venom (12.5–1.56 μg/mL) to PRP and incubating at 37 °C. Afterwards, adenosine diphosphate (ADP, 5 μM) was added, the baseline was set to 0, and the aggregation level was recorded for another 5 min. To measure platelet activation by flow cytometry, PRP was incubated with different concentrations of *N. nivea* venom (50–1.56 μg/mL) and 35 μL of an antibody master mix. The mastermix contained 2 μL each of P-selectin (PE-Cy™5 labelled mouse anti-human CD62P) and FITC-labelled fibrinogen (polyclonal rabbit anti-human fibrinogen, 2 μg/mL) antibodies and 31 μL of HEPES-buffered solution (containing 2.9 mM KCl, 134 mM NaCl, 0.34 mM Na_2_HPO_4_·12H_2_O, 1 mM MgCl_2_, 12 mM NaHCO_3_, 20 mM HEPES; pH 7.3). After incubating all samples for 5 min, the agonists ADP (5 μM), thrombin receptor-activating peptide-6 (TRAP-6, 10 μM), or U46619 (4 μM), a thromboxane A_2_ (TXA_2_) analogue, were added to the relevant samples. Then, samples were incubated for an additional 20 min at 37 °C. Before flow cytometry analysis (using a BD Accuri C6, Wokingham, UK), samples were fixed with 0.2% (*v*/*v*) formyl saline [0.2% formaldehyde in 0.9% (*w*/*v*) NaCl]. During analysis, 5000 cells from each sample were recorded to determine median fluorescence intensity. To evaluate the effect of venom treatment, all samples were normalised to the agonist-only controls, which were set at 100%.

### 4.10. Haemolytic Assay

After removing PRP from centrifuged whole human blood, human erythrocytes were collected from the bottom fraction of the vacutainers, which were dense with red blood cells (RBCs). To wash these erythrocytes, an equal volume of PBS was added to the tube, and the mixture was centrifuged at 2000× *g* for 2 min; the supernatant was then discarded. Following the washing process, the erythrocytes were resuspended in PBS and treated with *N. nivea* venom (50 μg/mL). After incubating the samples at different time points (0, 1, 6, and 24 h), they were centrifuged at 2000× *g* for 2 min, and 50 μL of the supernatant was transferred to a 96-well plate. Finally, absorbance was measured at 540 nm using a Fluostar Optima (BMG Labtech, Ortenberg, Germany) spectrofluorometer. To assess the impact of PLA_2_ and MP inhibition on venom-induced haemolysis, the venom (50 μg/mL) was incubated with varespladib (100 µM), prinomastat (100 µM), and a combination of both inhibitors (100 µM of each) for the same time intervals prior to analysis. All subsequent steps were performed as described above. In this experiment, PBS and 1% (*v*/*v*) Triton X-100 (Sigma-Aldrich, Gillingham, UK) were used as negative (NC) and positive (PC) controls, respectively.

### 4.11. Fibrinogenolytic Assay

*N. nivea* venom (100 μg/mL) was added to human fibrinogen (1 mg/mL; Sigma-Aldrich, Gillingham, UK) in PBS at an approximate ratio of 1:19 (venom:fibrinogen), and the samples were incubated at 37 °C for various time intervals (0, 1, 3, 6, 9, 24 h). At each time point, 30 μL of each sample was taken and immediately mixed with 2× reducing sample treatment buffer (2× RSTB) (40% [*w*/*v*] SDS, 10% [*v*/*v*] β-mercaptoethanol, 20% [*v*/*v*] glycerol, and 10% stacking gel buffer in nanopure water, along with a trace of bromophenol blue as a tracking dye), then boiled at 90 °C for 10 min. Afterwards, SDS-PAGE was used to analyse each sample. A venom concentration of 100 μg/mL was used to ensure detectable fibrinogen degradation in the SDS–PAGE analysis. The fibrinogen concentration was selected to ensure clear detection of proteolytic degradation products in the SDS–PAGE analysis.

### 4.12. Sodium Dodecyl Sulfate-Polyacrylamide Gel Electrophoresis (SDS-PAGE)

To perform gel electrophoresis, SDS-PAGE gels (12%; 10-well) were hand-casted as outlined in the following protocol: resolving gel containing acrylamide–bisacrylamide solution (30% [*w*/*v*], 14 mL), SDS (10% [*w*/*v*], 350 μL), resolving gel buffer (3 M Tris-HCl, pH 8.8; 4.38 mL), water (14.525 mL), ammonium persulfate (APS, 1.5% [*w*/*v*], 1.75 mL) and tetra-methyl-ethylene-diamine (TEMED; 34 μL); the stacking layer is made up of acrylamide–bisacrylamide solution (30% [*w*/*v*], 1.35 mL), SDS (10% [*w*/*v*], 100 μL), stacking gel buffer (0.5 M Tris-HCl, pH 6.8; 2.5 mL), water (5.75 mL), APS (1.5% [*w*/*v*], 500 μL) and TEMED (8 μL). For SDS-PAGE analysis, samples from the fibrinogenolytic assay were prepared by mixing 30 μL of each reaction mixture with an equal volume of 2× RSTB. Samples were heated for 10 min at 90 °C. This was followed by vortexing the samples and loading 30 μL of them onto the gel. Precision Plus Protein™ Dual Colour Standards (5 μL; Bio-Rad, Watford, UK) were used as the molecular mass reference. The run took 2 h at 70 volts using a Mini-PROTEAN Electrophoresis System (Bio-Rad, Watford, UK). Then, Coomassie blue [0.1% (*w*/*v*) Coomassie Brilliant Blue in 40% methanol and 10% acetic acid] was used to stain the subsequent gels for 2 h. Then, the gels were de-stained on a rocker for 2 h using a de-staining solution [10% (*v*/*v*) methanol and 10% (*v*/*v*) acetic acid in double-distilled water].

### 4.13. Venom Fractionation

Reversed-phase high-performance liquid chromatography (RP-HPLC; Spectra-Physics P200) was employed to separate *N. nivea* venom using a BDS Hypersil C18 column (250 × 4.6 mm) (Thermo Scientific, Loughborough, UK). An amount of 200 μL of 0.1% [*v*/*v*] trifluoroacetic acid (TFA) in water was used to dissolve the lyophilised venom (2 mg). After centrifuging the sample (8000× *g* for 5 min), 200 μL of the supernatant was manually injected into the instrument. Fractions were collected at a flow rate of 1 mL/min over 100 min, employing a staged gradient of buffer A (0.1% TFA [*v*/*v*] in HPLC water) and buffer B (0.1% TFA [*v*/*v*] in acetonitrile), with buffer B reaching 60% at almost 70 min into the process. Fractions were detected at 215 nm, and those with an absorbance exceeding 0.05 were collected and stored in the freezer for subsequent analysis. Collected fractions were lyophilised and reconstituted in 100 μL PBS. For SDS-PAGE analysis, 20 μL of each fraction was loaded onto the gel for comparative profiling without prior standardisation of protein concentration. In addition, fractions were screened for biological activity by flow cytometry, in which 5 μL of each fraction was used to assess platelet activation. These analyses were performed as an initial screening step prior to protein quantification, allowing identification of fractions with both biological activity and sufficient abundance for further characterisation.

### 4.14. Mass Spectrometry Analysis

A prominent protein band from the purified fraction was excised from the Coomassie-stained SDS-PAGE gel and sent to Alta Bioscience (Birmingham, UK) for mass spectrometry analysis. Trypsin digestion was performed using 10 μL of eluted samples (up to 10 μg of protein), to which 40 μL of 100 mM ammonium bicarbonate (pH 8) and 50 µL 10 mM dithiothreitol were added and incubated at 56 °C for 30 min. Samples were then cooled to room temperature and cysteines were alkylated by adding 50 µL of 50 mM iodoacetamide, mixing and incubating at room temperature in the dark for 30 min. Finally, 50 µL of trypsin gold (Promega, Southampton, UK; 6 ng/µL) was added to the samples and incubated at 37 °C overnight. The samples were then desalted using millipore C18 ZipTips (Merck KGaA, Darmstadt, Germany). The tips were prepared by pre-wetting in 100% acetonitrile and rinsed in 2 × 10 µL 0.1% formic acid. Samples were repeat pipetted throughout the volume of the samples ten times. The tip was then washed with 3 × 10 µL 0.1% formic acid to remove excess salts before elution of peptides with 10 µL of 50% acetonitrile/water/0.1% formic acid. Samples were dried down to remove the acetonitrile, and then re-suspended in 0.1% formic acid solution in water.

The peptides were concentrated and separated using a UltiMate^®^ 3000 HPLC (Dionex, Sunnyvale, CA, USA). Samples were trapped on precolumn, Acclaim PepMap 100 C18 HPLC Columns (Thermo Scientific, Loughborough, UK), 3 µm particle size, 2 cm length, 75 µm I.D., (Dionex, Sunnyvale, CA, USA) and separated in Nano Series™ standard solumns 75 µm I.D. × 15 cm, packed with C18 PepMap100, 2 µm, 100 Å (Dionex, Sunnyvale, CA, USA) using a gradient of 3.2% to 44% solvent B (0.1% formic acid in acetonitrile) for 30 min. The column was then washed with 90% mobile phase B before re-equilibrating at 3.2% mobile phase B. Peptides were eluted directly (~300 nL/min) via a Triversa Nanomate nanospray source (Advion Biosciences, Ithaca, NY, USA) into a QExactive HF Orbitrap mass spectrometer (ThermoFisher Scientific, Loughborough, UK). The spray voltage of QE HF was set to 1.7 kV through Triversa NanoMate and heated capillary at 275 °C. The mass spectrometer performed a full MS scan (*m*/*z* 360–1600) and subsequent HCD MS/MS scans of the 20 most abundant ions with dynamic exclusion setting 15S. Full-scan mass spectra were recorded at a resolution of 120,000 at *m*/*z* 200 and AGC target of 3 × 10^6^. Precursor ions were fragmented in HCD MS/MS with resolution set up at 15,000 and a normalised collision energy of 28. The width of the precursor isolation window was 1.2 *m*/*z* and only multiply charged precursor ions were selected for MS/MS.

The MS and MS/MS scans were searched against Uniprot database using Protein Discovery 2.2 software, Sequest HT algorithm (Thermo Fisher, Loughborough, UK). Variable modifications included were deamidation (N and Q), oxidation (M), phosphorylation (S, T and Y) and acetylation (K). The precursor mass tolerance was 10 ppm and the MS/MS mass tolerance was 0.02 Da. Two missed cleavages were allowed and data were filtered with a false discovery rate (FDR) of 0.01. Proteins with at least two high-confidence peptides were accepted as real hits.

### 4.15. Protein Quantification

Following venom fractionation, the protein concentration of selected venom fractions was determined using a NanoDrop 2000 spectrophotometer (Thermo Fisher Scientific, Waltham, MA, USA). Fractions were dissolved in PBS, with PBS used as the blank. A bovine serum albumin (BSA) standard (2 mg/mL) was serially diluted to generate a standard curve for protein quantification. For each measurement, 2 μL of each sample was loaded onto the NanoDrop pedestal, and measurements were performed in triplicate.

### 4.16. Lactate Dehydrogenase Cytotoxicity Assay

LDH assays were performed using an LDH cytotoxicity assay kit (Thermo Fisher Scientific, Loughborough, UK), as directed by the manufacturer. Washed platelets were incubated with *N. nivea* venom (50 μg/mL) for 45 min at 37 °C in a 96-well plate. Subsequently, 25 μL of the supernatant was transferred to a new 96-well plate, followed by the addition of 25 μL of the reaction mixture. Samples were incubated for 30 min at 37 °C in the dark, after which 25 μL of stop solution was added. Absorbance was then recorded at 490 nm and 680 nm using a Fluostar Optima (BMG Labtech, Ortenberg, Germany) spectrofluorometer. To measure the LDH activity of AB1190 cells, cells were seeded in a 96-well tissue culture plate (100 μL of growth media/well) and incubated at 37 °C with 5% CO_2_. After replacing the media with fresh growth media, *N. nivea* venom (50 μg/mL) or its purified toxin were added to each well and incubated for 4 h. Afterwards, 25 μL of each sample was transferred to a new 96-well plate, and the remaining steps of the experiment were carried out as described above. Triton X-100 (1%; Sigma, Bilston, UK) was used as the positive control. PBS and the cell culture media served as negative controls for the washed platelets and AB1190 cells cytotoxicity assay, respectively.

### 4.17. Cell Viability Assay

To investigate the impact of *N. nivea* venom on the viability of AB1190 myoblasts, an MTS cell viability assay kit (Promega, Southampton, UK) was used. Cells (5000 per well) were seeded in a 96-well plate containing 100 μL growth media and incubated overnight to allow cell adhesion. The growth media were replaced with fresh growth media, after which *N. nivea* venom (50 μg/mL) and or its purified cytotoxin (50 μg/mL) was added to each well and incubated for 24 h. Following the 24-h incubation, 10 μL MTS reagent was added to each well and incubated further for 4 h at 37 °C with 5% CO_2_. Absorbance was measured at 490 nm wavelength using Fluostar Optima (BMG Labtech, Ortenberg, Germany) spectrofluorometer. All experiments were performed in triplicate for each condition. Fresh growth media were used for the negative control, and Triton X-100 as a recognised cytotoxic agent (positive control). For analysis, each reading was compared to the negative control, and the percentage cell viability was calculated.

### 4.18. Statistical Analysis

Statistical analyses were performed using GraphPad Prism (version 7.0; GraphPad Software Inc., San Diego, CA, USA). Differences between multiple groups were analysed using ordinary one-way analysis of variance (ANOVA) followed by Fisher’s least significant difference (LSD) post hoc test. For comparisons between two groups (venom-treated samples and control samples), unpaired Student’s *t*-tests were performed. Data are presented as mean ± standard deviation (SD), and differences were considered statistically significant at *p* < 0.05.

## Data Availability

The original contributions presented in this study are included in the article/Supplementary Materials. Further inquiries can be directed to the corresponding author.

## Supplementary Materials

The following supporting information can be downloaded at: https://www.mdpi.com/article/10.3390/toxins18050211/s1. Figure S1: Haemolytic activity of *N. nivea* venom.

## References

[1] Slagboom J., Kool J., Harrison R.A., Casewell N.R. (2017). Haemotoxic snake venoms: Their functional activity, impact on snakebite victims and pharmaceutical promise. Br. J. Haematol..

[2] Maduwage K., Isbister G.K. (2014). Current treatment for venom-induced consumption coagulopathy resulting from snakebite. PLoS Negl. Trop. Dis..

[3] Furie B., Furie B.C. (2008). Mechanisms of thrombus formation. N. Engl. J. Med..

[4] Hoffman M., Monroe D.M. (2001). A cell-based model of hemostasis. Thromb. Haemost..

[5] Park E.J., Choi S., Kim H.H., Jung Y.S. (2020). Novel Treatment Strategy for Patients with Venom-Induced Consumptive Coagulopathy from a Pit Viper Bite. Toxins.

[6] Moore G.W. (2022). Snake Venoms in Diagnostic Hemostasis and Thrombosis. Semin. Thromb. Hemost..

[7] Alvarez Flores M., Faria F., Andrade S., Chudzinski-Tavassi A. (2017). Snake Venom Components Affecting the Coagulation System.

[8] Bittenbinder M.A., Zdenek C.N., Op den Brouw B., Youngman N.J., Dobson J.S., Naude A., Vonk F.J., Fry B.G. (2018). Coagulotoxic Cobras: Clinical Implications of Strong Anticoagulant Actions of African Spitting Naja Venoms That Are Not Neutralised by Antivenom but Are by LY315920 (Varespladib). Toxins.

[9] Tasoulis T., Isbister G.K. (2017). A Review and Database of Snake Venom Proteomes. Toxins.

[10] Offor B.C., Muller B., Piater L.A. (2022). A Review of the Proteomic Profiling of African Viperidae and Elapidae Snake Venoms and Their Antivenom Neutralisation. Toxins.

[11] Dutta S., Gogoi D., Mukherjee A.K. (2015). Anticoagulant mechanism and platelet deaggregation property of a non-cytotoxic, acidic phospholipase A2 purified from Indian cobra (*Naja naja*) venom: Inhibition of anticoagulant activity by low molecular weight heparin. Biochimie.

[12] Chowdhury A., Lewin M.R., Zdenek C.N., Carter R., Fry B.G. (2021). The Relative Efficacy of Chemically Diverse Small-Molecule Enzyme-Inhibitors Against Anticoagulant Activities of African Spitting Cobra (*Naja* Species) Venoms. Front. Immunol..

[13] Tan C.H., Wong K.Y., Huang L.K., Tan K.Y., Tan N.H., Wu W.G. (2022). Snake Venomics and Antivenomics of Cape Cobra (*Naja nivea*) from South Africa: Insights into Venom Toxicity and Cross-Neutralization Activity. Toxins.

[14] Lüddecke T., Avella I., Damm M., Schulte L., Eichberg J., Hardes K., Schiffmann S., Henke M., Timm T., Lochnit G. (2024). The Toxin Diversity, Cytotoxicity, and Enzymatic Activity of Cape Cobra (*Naja nivea*) Venom. Toxins.

[15] Phelps T. (2007). Observations of the Cape cobra, *Naja nivea* (Serpentes: Elapidae) in the DeHoop Nature Reserve, Western Cape Province, South Africa. Herpetol. Bull..

[16] Nirthanan S., Gwee M.C. (2004). Three-finger alpha-neurotoxins and the nicotinic acetylcholine receptor, forty years on. J. Pharmacol. Sci..

[17] Wang C.I., Reeks T., Vetter I., Vergara I., Kovtun O., Lewis R.J., Alewood P.F., Durek T. (2014). Isolation and structural and pharmacological characterization of α-elapitoxin-Dpp2d, an amidated three finger toxin from black mamba venom. Biochemistry.

[18] Neumann N.R., du Plessis A., van Hoving D.J., Hoyte C.O., Lermer A., Wittels S., Marks C. (2023). Antivenom supply and demand: An analysis of antivenom availability and utilization in South Africa. Afr. J. Emerg. Med..

[19] Lafnoune A., Chbel A., Darkaoui B., Nait Irahal I., Oukkache N. (2024). Cobra Venom: From Envenomation Syndromes to Therapeutic Innovations. Int. J. Pept. Res. Ther..

[20] Kini R.M. (2005). The intriguing world of prothrombin activators from snake venom. Toxicon.

[21] Chuaikhongthong W., Khimmaktong W., Thipthong N., Lorthong N., Chaisakul J. (2025). Respiratory Muscle Injury Following Acute Monocled Cobra (*Naja kaouthia*) Envenoming: Histopathological Study in Rat Diaphragm. Curr. Issues Mol. Biol..

[22] McFarlane L.O., Pukala T.L. (2024). Proteomic Investigation of Cape Cobra (*Naja nivea*) Venom Reveals First Evidence of Quaternary Protein Structures. Toxins.

[23] UCSD (2024). Snakebite Protocols: Cape Cobra (Naja nivea).

[24] Chanda C., Sarkar A., Sistla S., Chakrabarty D. (2013). Anti-platelet activity of a three-finger toxin (3FTx) from Indian monocled cobra (*Naja kaouthia*) venom. Biochem. Biophys. Res. Commun..

[25] Kazandjian T.D., Arrahman A., Still K.B.M., Somsen G.W., Vonk F.J., Casewell N.R., Wilkinson M.C., Kool J. (2021). Anticoagulant Activity of *Naja nigricollis* Venom Is Mediated by Phospholipase A2 Toxins and Inhibited by Varespladib. Toxins.

[26] Zdenek C.N., Rodrigues C.F.B., Bourke L.A., Tanaka-Azevedo A.M., Monagle P., Fry B.G. (2023). Children and Snakebite: Snake Venom Effects on Adult and Paediatric Plasma. Toxins.

[27] Teixeira C., Fernandes C.M., Leiguez E., Chudzinski-Tavassi A.M. (2019). Inflammation Induced by Platelet-Activating Viperid Snake Venoms: Perspectives on Thromboinflammation. Front. Immunol..

[28] Gan Z.R., Gould R.J., Jacobs J.W., Friedman P.A., Polokoff M.A. (1988). Echistatin. A potent platelet aggregation inhibitor from the venom of the viper, Echis carinatus. J. Biol. Chem..

[29] Owen W.G., Walker F.J., Esmon C.T. (1982). Prothrombin activation by a protease from taipan snake venom. J. Biol. Chem..

[30] Rao V.S., Kini R.M. (2002). Pseutarin C, a prothrombin activator from Pseudonaja textilis venom: Its structural and functional similarity to mammalian coagulation factor Xa-Va complex. Thromb. Haemost..

[31] Larréché S., Chacha R.B., Sodjinou N., Ouorou S.A., Ganhouingnon E., Layo E.A., Mégarbane B., Massougbodji A., Chippaux J.P. (2024). Viscoelastic Study of Hemostasis Disorders Associated with Echis ocellatus Envenoming in North Benin Using a Quantra Analyzer. Toxins.

[32] Cheng A.-C., Wu H.-L., Shi G.-Y., Tsai I.-H. (2012). A Novel Heparin-dependent Inhibitor of Activated Protein C That Potentiates Consumptive Coagulopathy in Russell’s Viper Envenomation. J. Biol. Chem..

[33] Vu T.T., Stafford A.R., Leslie B.A., Kim P.Y., Fredenburgh J.C., Weitz J.I. (2013). Batroxobin binds fibrin with higher affinity and promotes clot expansion to a greater extent than thrombin. J. Biol. Chem..

[34] Kini R.M., Koh C.Y. (2020). Snake venom three-finger toxins and their potential in drug development targeting cardiovascular diseases. Biochem. Pharmacol..

[35] Barnwal B., Jobichen C., Girish V.M., Foo C.S., Sivaraman J., Kini R.M. (2016). Ringhalexin from *Hemachatus haemachatus*: A novel inhibitor of extrinsic tenase complex. Sci. Rep..

[36] Utkin Y.N. (2019). Last decade update for three-finger toxins: Newly emerging structures and biological activities. World J. Biol. Chem..

[37] Girish V.M., Kumar S., Joseph L., Jobichen C., Kini R.M., Sivaraman J. (2012). Identification and structural characterization of a new three-finger toxin hemachatoxin from Hemachatus haemachatus venom. PLoS ONE.

[38] Lazarovici P., Marcinkiewicz C., Lelkes P.I. (2019). From Snake Venom’s Disintegrins and C-Type Lectins to Anti-Platelet Drugs. Toxins.

[39] Layfield H.J., Williams H.F., Ravishankar D., Mehmi A., Sonavane M., Salim A., Vaiyapuri R., Lakshminarayanan K., Vallance T.M., Bicknell A.B. (2020). Repurposing Cancer Drugs Batimastat and Marimastat to Inhibit the Activity of a Group I Metalloprotease from the Venom of the Western Diamondback Rattlesnake, *Crotalus atrox*. Toxins.

[40] Vaiyapuri S., Harrison R.A., Bicknell A.B., Gibbins J.M., Hutchinson G. (2010). Purification and functional characterisation of rhinocerase, a novel serine protease from the venom of Bitis gabonica rhinoceros. PLoS ONE.

[41] Díaz-García A., Ruiz-Fuentes J.L., Yglesias-Rivera A., Rodríguez-Sánchez H., Riquenes Garlobo Y., Fleitas Martinez O., Fraga Castro J.A. (2015). Enzymatic analysis of venom from Cuban scorpion *Rhopalurus junceus*. J. Venom Res..

[42] Vaiyapuri S., Jones C.I., Sasikumar P., Moraes L.A., Munger S.J., Wright J.R., Ali M.S., Sage T., Kaiser W.J., Tucker K.L. (2012). Gap junctions and connexin hemichannels underpin hemostasis and thrombosis. Circulation.

[43] Senthilkumaran S., Patel K., Rajan E., Vijayakumar P., Miller S.W., Rucavado A., Gilabadi S., Sonavane M., Richards N.J., Williams J. (2023). Peripheral Arterial Thrombosis following Russell’s Viper Bites. TH Open.

