# Supplementary material for: Effects of Cape Cobra (Naja nivea) Venom and Its Isolated Protein on the Modulation of Platelet Activation
Source: Toxins (Basel). 2026 Apr 30;18(5):211. doi: 10.3390/toxins18050211 (PMC13211613; doi:10.3390/toxins18050211)
Supplement: Supplementary file 1 [file toxins-18-00211-s001.zip › toxins-4207230-supplementary.pdf]

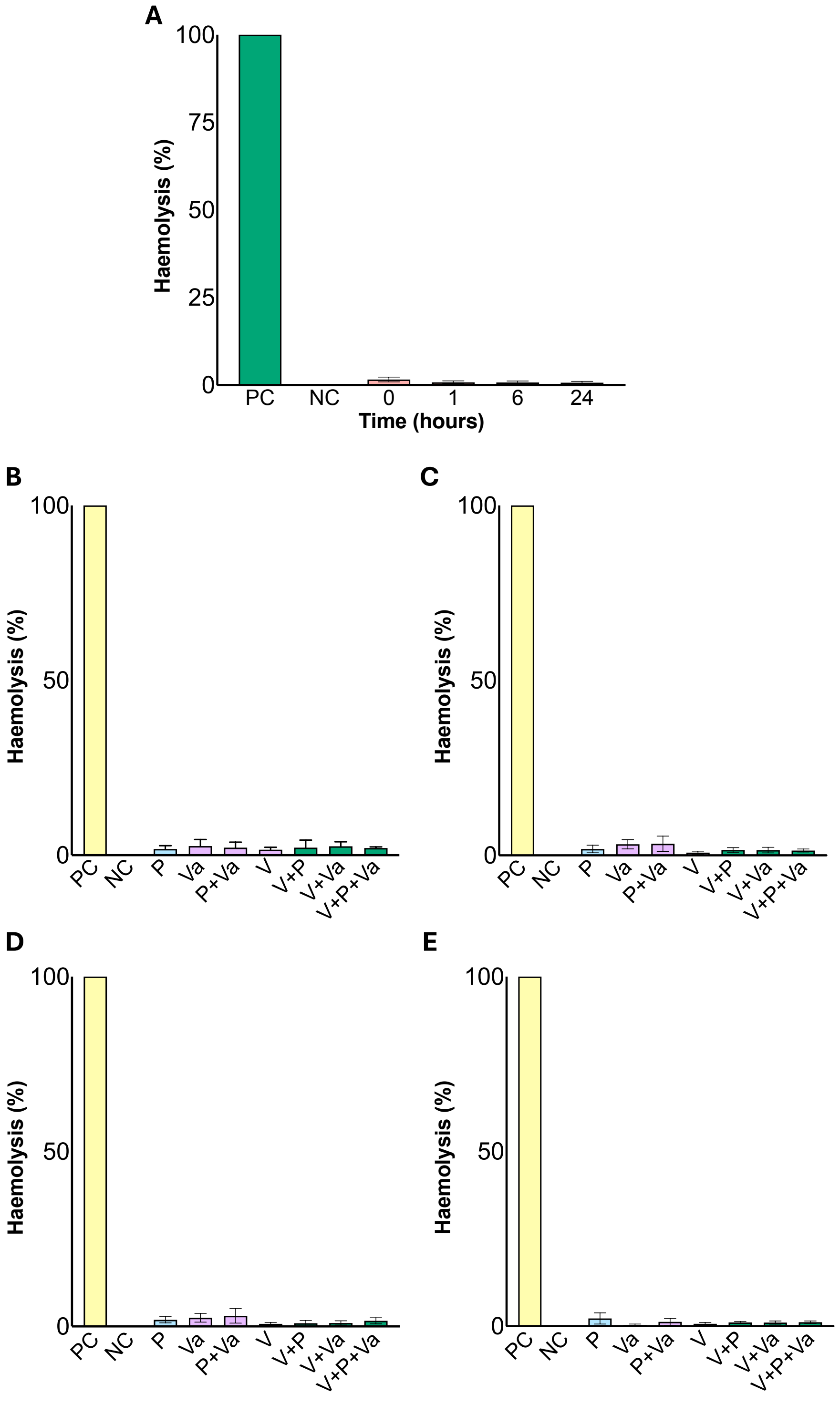

Figure S1: Haemolytic activity of *N. nivea* venom. (A) Haemolytic activity caused by the venom (50 μg/mL) on washed human red blood cells (RBC) was measured at four different time points (0,1,6, and 24 hours). The impacts of prinomastat and varespladib (100 μg/mL) on venom-induced blood haemolysis were monitored at 0 h (B), 1h (C), 6 h (D), and 24 h (E). A detergent (1% of Triton X-100; Sigma, UK) was used as the positive control (PC) and was considered as 100% haemolysis, while PBS was used as the negative control (NC). All samples were normalised against the PC, and results are given as haemolysis (%). For statistical comparison, venom alone (V) was compared to the NC, and inhibitor-treated samples were compared to V. Data are presented as mean ± SD (n = 4). One-way ANOVA followed by Fisher's LSD test was used for statistical analysis.
